# Measurements of Iodination in Thyroglobulin: A Step Toward the Next Generation of Thyroid Cancer Monitoring

**DOI:** 10.1210/jendso/bvaf015

**Published:** 2025-01-21

**Authors:** Anthony Maus, Chris Thompson, Stefan K G Grebe

**Affiliations:** Department of Laboratory Medicine and Pathology, Division of Clinical Biochemistry and Immunology, Mayo Clinic, Rochester, MN 55905, USA; Department of Laboratory Medicine and Pathology, Division of Clinical Biochemistry and Immunology, Mayo Clinic, Rochester, MN 55905, USA; Department of Laboratory Medicine and Pathology, Division of Clinical Biochemistry and Immunology, Mayo Clinic, Rochester, MN 55905, USA; Department of Laboratory Medicine and Pathology, Division of Laboratory Genetics and Genomics, Mayo Clinic, Rochester, MN 55905, USA; Department of Medicine, Division of Endocrinology, Mayo Clinic, Rochester, MN 55905, USA

**Keywords:** thyroglobulin, bottom-up proteomics, LC-MS/MS

## Abstract

Thyroglobulin (Tg) is a 330-kDa homodimeric protein that that is the prohormone of thyroid hormones triiodothyronine (T3) and thyroxine (T4). The most critical steps of thyroid hormone synthesis by Tg are iodination and fusion of specific tyrosine residues that are in close proximity to each other in the folded Tg protein. The degree of Tg iodination has been studied widely to determine if it is correlated with thyroid autoimmune disease with mixed results, but these efforts have been limited by the lack of an effective quantitative technique. Simultaneously, the treatment of thyroid cancer has undergone a shift toward partial thyroidectomies, thus undermining the value of Tg measurements. A possible alternative to established monitoring techniques is measurement of Tg iodination states as it has been shown that tumor-derived Tg has significantly lower iodine content. Such measurements require a thorough understanding of normal iodination status. In this study, state-of-the-art liquid chromatography–tandem mass spectrometry (LC-MS/MS) instrumentation is used to perform bottom-up proteomics experiments and identify iodinated residues within commercially available Tg. Using this technique, sequence coverages greater than 90% were achieved, which resulted in identification of previously identified and novel hormone synthesis and donor sites. Based on the results of these discovery experiments, 5 iodination sites were selected for targeted quantitative LC-MS/MS measurements, which suggested that hormone synthesis occurs predominantly at Y24 and Y2766. The results presented herein lay the foundation for routine measurements of iodinated residues, which has the potential to overcome the limitations of current monitoring techniques and benefit patient care.

Thyroid cancer is relatively common, with the American Cancer Society projecting 44 020 new cases in 2024 in the United States alone [1]. The disease disproportionately affects females, with approximately 3 out of every 4 patient cases being female, making it the fifth most common cancer in women [2]. However, despite the relatively high incidence rate, prognosis is generally good [3]. Immediate treatment is not necessary in all cases, but surgery is the most common treatment when warranted [4, 5]. When surgery is performed, the extent of resection is a key decision that remains controversial and affects the probability of recurrence and postsurgery treatment and monitoring [6, 7].

After surgery, monitoring for recurrence of the disease is often necessary, with serum thyroglobulin measurements being considered essential in many cases [8, 9]. Thyroglobulin (Tg) is a 330-kDa protein that forms homodimers on reaching conformational maturation [10]. The primary role of Tg is storage of iodine and synthesis of the thyroid hormones triiodothyronine (T3) and thyroxine (T4) [10, 11]. Despite the well-established role of Tg in disease monitoring, there are limitations to current testing techniques. High-sensitivity assays are imperative for detecting residual and recurrent disease [12]. Several immunoassays are commercially available that are capable of sufficient sensitivity, but this methodology is prone to interferences from autoantibodies against Tg [12-14] and heterophile antibodies [15, 16]. Liquid chromatography–tandem mass spectrometry (LC-MS/MS) techniques overcome these limitations, but matching the sensitivity of immunoassays with MS requires complicated sample-preparation protocols and high-performance mass spectrometers [17, 18]. Additionally, identifying an ideal calibration that allows for harmonization of LC-MS/MS testing has been challenging [18, 19]. Regardless of the testing methodology used, it is very difficult to monitor for disease recurrence following a partial resection because the level of Tg in blood varies depending on the amount of residual tissue and there are currently no reference intervals for these patients [20]. An alternative approach to Tg testing could be analysis of the level of iodination of histidine and tyrosine residues within Tg. It has been shown that Tg derived from carcinomas has very low iodine content and is not iodinated to the extent necessary to form thyroid hormones [21, 22]. A probable reason why tumor-derived Tg might be poorly iodinated is that the harsh redox environment necessary for the iodination would likely destroy thyrocytes; hence nature has relegated iodination to the thyroid follicles. Since most thyroid cancers do not form functional follicles, any Tg made by tumor cells might lack iodination.

There are several additional potential applications of Tg iodination measurements. Insufficient iodine intake is known to increase the prevalence of goiters and neonatal hypothyroidism. For example, Tg iodination measurements could help differentiate between causes of congenital hypothyroidism and neonatal hypothyroidism due to insufficient iodine intake [23]. Alternatively, excessive iodoprophylaxis has been shown to have negative implications such as increased prevalence of hypothyroidism and thyroid autoimmunity [24, 25]. The increased rate of thyroid autoimmunity is likely due to iodine-containing neoantigenic determinants that elicit an autoimmune response, and this phenomenon has been linked to thyroid disfunction and papillary thyroid carcinoma [25-27]. Therefore, direct measurements of Tg iodination could be used to mitigate the implications of insufficient or excessive iodoprophylaxis. By providing an assessment of thyroid hormone synthesis potential, Tg iodination measurements could also be used to provide evidence of factitious hyperthyroidism due to excessive intake of thyroid hormones by assessing thyroid synthesis potential, which is difficult to diagnose using currently available laboratory techniques [28, 29].

However, to fully use iodination as a testing modality, a thorough understanding of Tg iodination is necessary and a method capable of performing quantitative Tg iodination measurements must be developed. In 1989, Lamas and colleagues [30] used ^125^I labeling, tryptic digestion, high-performance LC fractionation, and amino acid sequencing to build an iodination map. However, modern LC-MS/MS instrumentation is ideally suited for analysis of posttranslational modifications such as iodination. Dedieu et al [31] demonstrated this in 2010 when analyzing Tg derived from mice. Using a bottom-up proteomics approach, they identified iodination sites and 2 potential donor sites based on detection of pyruvic acid modifications and corresponding peptide cleavages. Coscia and colleagues [32] further explored the relationship between donors and acceptor sites in the formation of thyroid hormones using cryo-electron microscopy and determined that there were likely 4 acceptor and 5 donor tyrosine residues in Tg. A limitation of all of these noteworthy iodination studies is a lack of quantitative analysis of the iodination states. In our study, we build on this previous work and use hybrid orbitrap instrumentation to update the map of iodination within human Tg. Based on the results from our discovery experiments and the published studies, 5 iodination sites were selected for targeted measurements using stable isotope–labeled synthetic peptides [33, 34], which enables comparison of the abundance of the different iodination states and positions.

## Materials and Methods

### Chemicals and Reagents

Isolated thyroglobulin was purchased from Cell Sciences (catalog No. CSI14829A) and Millipore-Sigma (catalog No. 609312-1MG). These materials were tested on our clinical LC-MS/MS Tg test [14], which is traceable to the Beckman Access Thyroglobulin (Tg) Immunoassay (Beckman Coulter, catalog No. A36920, RRID:AB_3674086), and determined to be 68.2% and 67.4% pure by mass, respectively. Trypsin was purchased from Worthington Biochemical Corporation (catalog No. 2744), chymotrypsin was purchased from ThermoFisher Scientific (catalog No. 90056), and GluC was purchased from Millipore-Sigma (catalog No. 11047817001). Water for reagent preparation was purified using a Milli-Q IQ 7000. Zwittergent Z3-16 was purchased from CalBiochem (EMD Millipore). Tris base, hydrochloric acid, and trifluoroacetic acid (TFA) were purchased from ThermoFisher Scientific. Ammonium bicarbonate, dithiothreitol (DTT), and iodoacetamide (IAA) were purchased from Millipore-Sigma.

### Enzymatic Digestion of Thyroglobulin

Tg isolates were suspended in water with 0.002% Z3-16 at a concentration of 1 mg/mL based on the manufacturer's stated mass. The digestion buffer was 100 mM Tris-HCl pH 8.0 for trypsin and chymotrypsin, and 100 mM ammonium bicarbonate pH 7.8 for GluC digestion. Thyroglobulin isolates (20 μL) were spiked into 180 μL of appropriate digestion buffer. Next, 20 μL of 100 mM DTT was added and samples were incubated for 40 minutes at 48 °C followed by addition of 50 μL of 100 mM IAA and incubation in the dark for 30 minutes at room temperature. Enzymes were added at a 1:20 enzyme:protein ratio by mass. Enzymatic digestion was performed at 37 °C for 15 hours. The digestion was stopped by bringing the solution to 0.5% TFA. Six digests were performed using each enzyme for both source materials.

### Liquid Chromatography–Tandem Mass Spectrometry Analysis for Discovery Experiments

LC separation was performed with an EvoSep One LC system, which used standardized preformed gradients. Prior to LC separation, 200 μL of the digest was loaded offline onto Evotip Pure reversed-phase disposable trap columns, which enable sample loading and desalting while minimizing carryover. The LC separation was performed using the preformed “extended method” (88 minutes gradient) and the EV1109 column. Mobile phase A was water with 0.1% formic acid (BJLC452-2.5) and mobile phase B was acetonitrile with 0.1% formic acid (BJLC441-2.5), both of which were purchased from VWR Scientific. Data-dependent tandem MS analysis was performed using an Thermo Scientific Exploris 480 high-resolution tandem MS. The Exploris 480 was equipped with an EasySpray electrospray ionization source that was operated at 2100 V and the ion transfer tube temperature was 285 °C. MS scans were conducted from m/z 350 to 1600 at a resolution of 120 000. The maximum fill time was 200 ms, the normalized automatic gain control target was 300%, and the radiofrequency lens was set to 50%. Tandem MS was then initiated on the top 12 precursor ions with charge states 2 to 5 and a dynamic exclusion of 25 seconds. MS/MS was performed with a normalized higher collisional dissociation collision energy of 28%, resolution of 30 000, maximum fill time of 55 seconds, and a normalized automatic gain control target of 200%.

### Data Processing for Discovery Experiments

Database searching was performed using Proteome Discoverer v. 2.5 against the UniProt human protein database and common MS contaminants. The RAW files from the 6 replicates were combined for database searching. The search parameters included a maximum number of 2 missed cleavages and carbamidomethylation at cysteine as a fixed modification. Variable modifications included methionine oxidation of cysteine, phosphorylation, iodination (I, + 125.8966), diiodination (I2,+251.7933), triiodothyronine (T3, + 469.7162), and thyroxine (T4, + 595.6128), dehydroalanine (−94.0419), and pyruvate modification (−91.0422) of tyrosine.

### Sample Preparation for Target Quantitation Using Stable-Isotope Labeled Peptides

Stable-isotope–labeled peptides for selected iodination sites were synthesized at the Mayo Clinic Proteomics Core. “Winged” peptides [35] with 5 amino acids on each side of the cleavage sites without iodination (NoI), I, I2, T3, and T4 were synthesized for each target iodination site. Details on selected target peptides such as amino acid sequence, optimal enzyme, and precursor ion masses can be seen in Supplementary Table S1 [36]. After synthesis, amino acid analysis was performed to determine the concentration of each amino acid residue by Creative Proteomics. Based on the concentrations determined by amino acid analysis, a stock solution was prepared that enabled addition of 2 pmol of NoI, I, and I2 and 20 pmol of T3 and T4 by spiking 20 μL from the stock solution. This stock was added to 20 μL of 1 mg/mL Tg. Next, 60 μL of buffer was added for each of the respective enzymes as described earlier. Reduction was then performed by adding 10 μL of 100 mM DTT and incubating at 48 °C for 40 minutes. This was followed by alkylation, which required addition of 25 μL of 100 mM IAA and incubation at room temperature in the dark for 30 minutes. As described above, enzymes were added at a 1:20 enzyme:protein ratio by mass and digestion was performed at 37 °C for 15 hours. The digestion was stopped by bringing the solution to 0.5% TFA. Three replicates were prepared for each enzyme and source material, which resulted in 18 total samples digested.

### Liquid Chromatography–Tandem Mass Spectrometry Analysis for Target Quantitation Using Stable-Isotope Labeled Peptides

LC separation was performed using a Thermo Scientific Vanquish Neo UHPLC system. Mobile phases were the same as described earlier. Peptides were injected (25 μL) and loaded onto an Acclaim PepMap 100 C18 trap column (catalog No. 164199) at 1% B. Peptides were separated using an EV1109 column and a 400 nL/min flow rate. The gradient ramped from 1% B to 55% B over 80 minutes, was then ramped to 98% B over 5 minutes, was held at 98% B for 5 minutes, then jumped back to 1% B and equilibrated at starting conditions. Data-dependent tandem MS was again conducted using an Exploris 480 operated as described earlier.

### Data Processing for Target Quantitation Using Stable-Isotope Labeled Peptides

Data were processed using TraceFinder v. 5.1. (Thermo Scientific). Extracted ion chromatograms were generated for target peptides by summing the 3 most abundant precursor ion isotopes within a mass tolerance of 20 ppm. Chromatographic peaks within the extracted ion chromatograms were integrated. Peak areas were exported to excel for further processing and determination of calculated concentrations.

## Results

**Figure 1.**
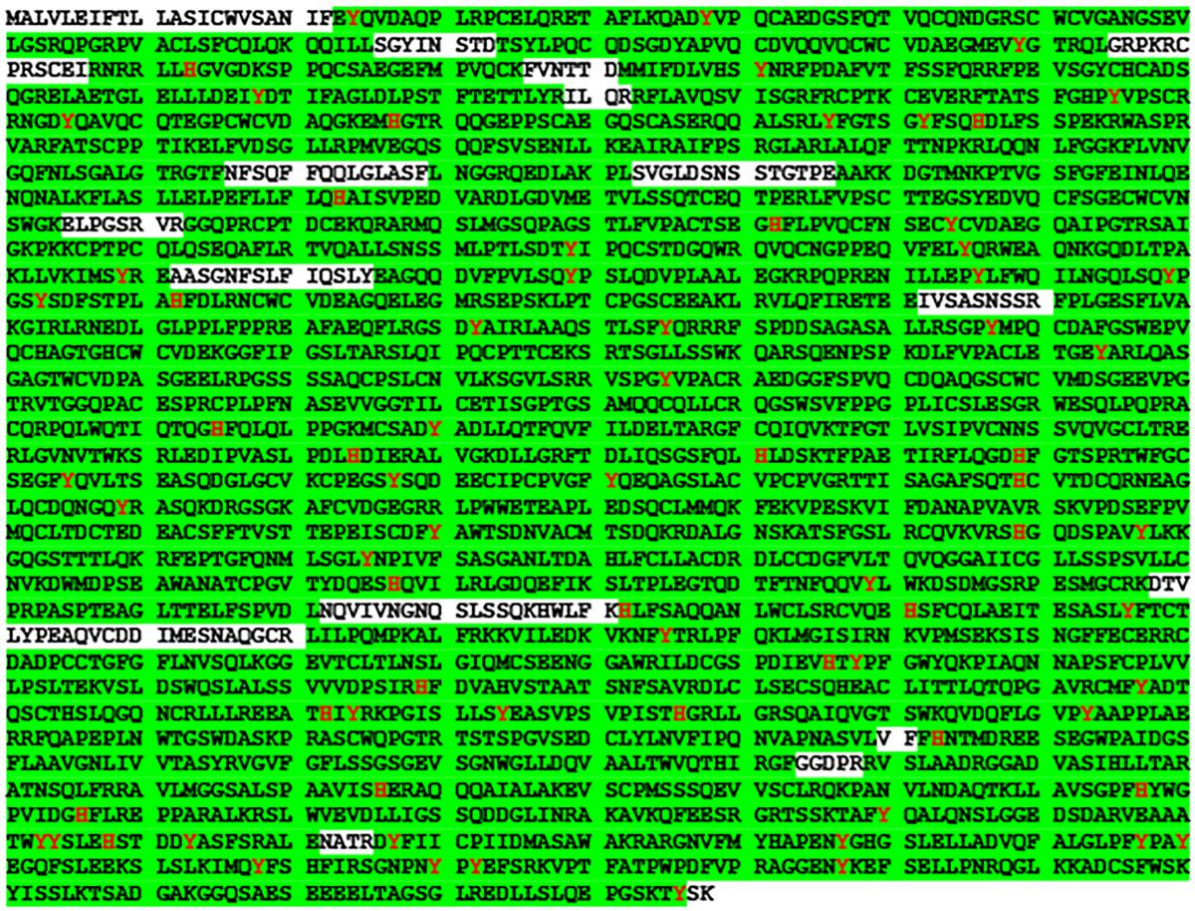
Sequence coverage of thyroglobulin sourced from cell sciences when combining data derived from the 3 enzymes used in this study. Highlight indicates identified residues and red indicates that iodination was detected at that site.

The methodology presented herein resulted in an exceptional percentage sequence coverage of Tg, with more than 90% observed when combining the results from the 3 enzymes used in this study (Table 1). A visual representation of the combined percentage sequence coverage of Tg for the Cell Sciences and Millipore-Sigma source material is shown in Fig. 1 and Supplementary Fig. S1 [36], respectively. In this figure, green highlights indicate regions that were identified by database searching of the LC-MS/MS data and red amino acids are those that exhibited iodination. As the numerical data presented in Table 1 suggest, nearly the entire Tg protein was detected through the course of this investigation.

**Table 1.** Percentage sequence coverage of thyroglobulin from the different sources and enzymes used in this study

Enzyme	Cell Sciences	Millipore-Sigma
GluC	71.7%	73.9%
Trypsin	78.0%	72.9%
Chymotrypsin	80.6%	79.9%
Combined	94.0%	91.9%

Identified peptides with iodinated residues from GluC, trypsin, and chymotrypsin are shown in Supplementary Tables S2 to S4 [36]. These tables show the extent of iodination, which includes monoiodination, diiodination, triiodination/T3 precursor, and tetraiodiation/T4 precursor. With the GluC enzyme, 34 iodinated amino acids were detected for the Cell Sciences Tg, whereas only 19 were detected for the Millipore-Sigma Tg. For the tryptic digests, 56 iodinated amino acids were observed for Cell Sciences Tg and 45 were observed for Millipore-Sigma Tg. Similarly, 51 iodinated residues were identified for Cell Sciences Tg and 32 were identified for Millipore-Sigma when chymotrypsin was used. Overall, when combining information from all of the enzymes, 54 of 66 (82%) total tyrosine amino acids and 23 of 39 (59%) total histidine amino acids were determined to be iodinated to some extent in the Cell Sciences Tg. The Millipore-Sigma material was not iodinated to the same extent, with 47 of 66 (71%) tyrosine amino acids iodinated and 16 of 39 (41%) histidines iodinated. A summary of only the T3 and T4 precursors detected are shown in Table 2, including the results from the Lamas and colleagues [30] and Coscia et al [32] for comparison. For the Cell Sciences material, 2 T3 precursor modifications were detected with both chymotrypsin and GluC, whereas 7 T3 precursor modifications were detected with trypsin. Only 1 T4 precursor was identified by GluC, 2 T4 precursors were identified with chymotrypsin, and 4 were identified with trypsin. Following the trend of less extensive iodination within the Millipore-Sigma material, only one T3 and T4 precursor was detected with GluC and trypsin. Using chymotrypsin, T3 precursor was detected in 2 positions and T4 precursor was again detected in only 1 position.

**Table 2.** Summary of tyrosine residues with triiodothyronine and thyroxine precursors detected

Analysis	Positions with T3 precursor	Positions with T4 precursor
Cell Sciences-GluC	Y24, Y2573	Y24
Cell Sciences-Trypsin	Y1310, Y2540, Y2563, 2564, Y2573, Y2670, Y2672	Y1310, Y2573, Y2670, Y2672
Cell Sciences-Chymotrypsin	Y2573, Y2766	Y2573, Y2766
Millipore-GluC	Y24	Y24
Millipore-Trypsin	Y2573	Y2573
Millipore-Chymotrypsin	Y2573, Y2766	Y2573
Lamas et al, 1989 [30]	24, 704, 2573, 2766	24, 704, 2573, 2766
Coscia et al, 2020 [32]	24, 1310, 2573, 2766	24, 1310, 2573, 2766

Abbreviations: T3, triiodothyronine; T4, thyroxine.

In addition to iodinated residues, these data were also used to search for donor tyrosine residues based on the tyrosine modifications yielding dehydroalanine or pyruvate. Identified dehydroalanine modifications are shown in Table 3. This modification was detected on 4 tyrosine residues. Additionally, the tyrosine to pyruvate conversion with subsequent peptide cleavage was observed at position Y2540 using the GluC enzyme. The database search yielded an identification of the sequence YQALQNSLGGEDSDARVE with a mass shift corresponding to the tyrosine to pyruvate conversion based on the work by Dedieu et al [31] and the corresponding expected N-terminal cleavage product (E)SRGRTSSKTAF.

**Table 3.** Dehydroalanine-modified tyrosine residues identified

Analysis	Position with Tyr->Dha	Peptide sequence
Cell Sciences Trypsin	Y234	RFPEVSG**Y**CHCADSQGR
Cell Sciences GluC	Y315	RFTATSFGHP**Y**VPSCRRNGDYQAVQCQTE
Cell Sciences GluC	Y325	RFTATSFGHPYVPSCRRNGD**Y**QAVQCQTE
Millipore-Sigma Trypsin	Y325	RNGD**Y**QAVQCQTEGPCWCVDAQGK
Millipore-Sigma Chymotrypsin	Y2157	QTQPGAVRCMF**Y**ADTQSCTHSL

Abbreviations: Dha, dehydroalanine; Tyr, tyrosine.

**Figure 2.**
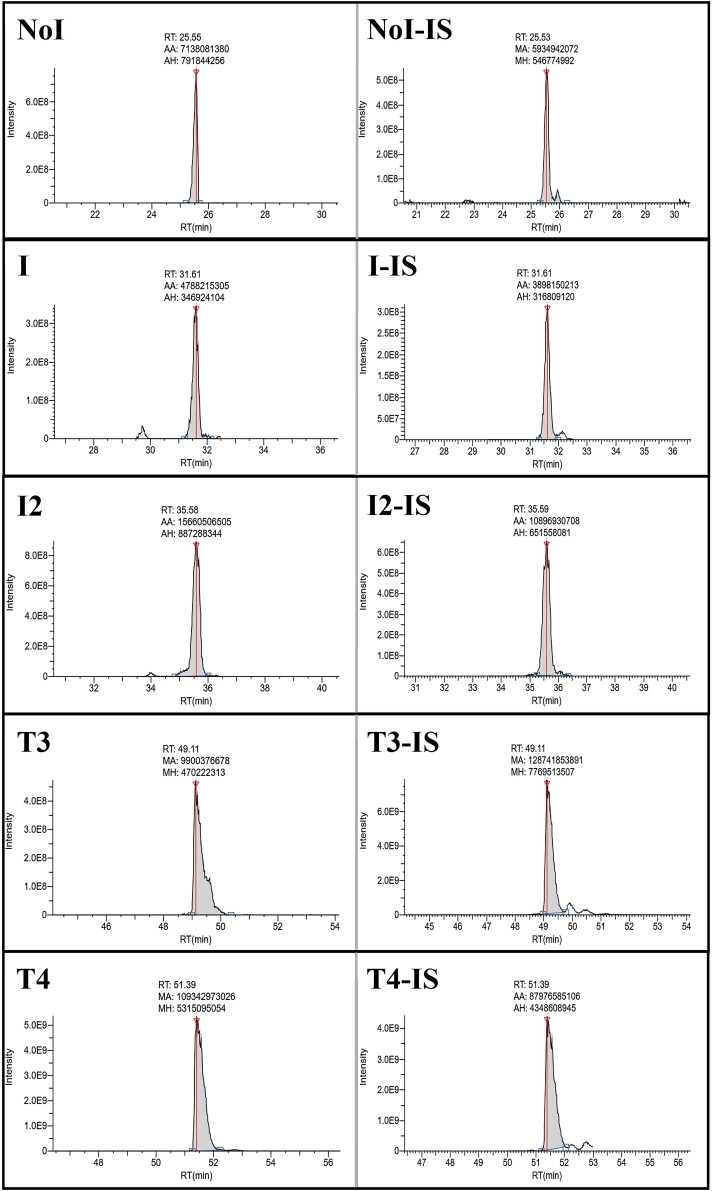
Representative chromatograms for the different iodination states of the Y2573 position. The isotopically labeled peptide is shown on the right and the unlabeled peptide is shown on the left for each iodination state.

**Figure 3.**
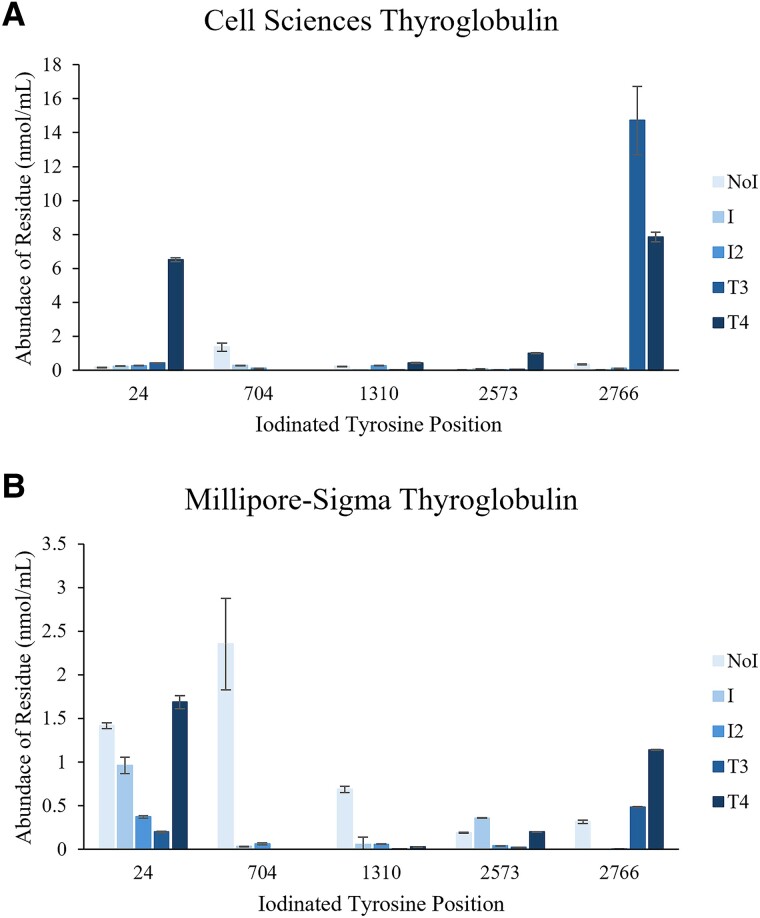
Results from stable isotope–labeled synthetic peptide quantitation experiments for the A, Cell Sciences thyroglobulin and B, Millipore-Sigma thyroglobulin. Each bar represents the mean and the error bars show ±1 SD (n = 3 replicates).

The LC-MS/MS identifications summarized in Table 2, a comparison of signal intensities from the identified peptides using the different enzymes shown in Supplementary Table S5 [36], and the iodination sites identified in relevant publications [30-32], target peptides, and coinciding enzymes for digestion, were selected. Based on these factors, 5 target iodination sites and peptides were selected for quantitation using stable isotope–labeled synthetic peptides. It was determined that GluC was optimal for Y24 and Y704, trypsin was best for Y1310, and chymotrypsin yielded the best results for Y2573 and Y2766. Representative chromatograms for the Y2573 position are shown in Fig. 2, and the other iodination sites quantitated are shown in Supplementary Figs. S2 to S5 [36]. Average results (n = 3) from the targeted quantitative measurement of the various iodination states for these positions are shown in Fig. 3. As indicated by the results from the discovery experiments, the Cell Sciences Tg (Fig. 3A) had a significantly higher concentration of iodinated residues than the Millipore-Sigma Tg (Fig. 3B). The tyrosines at positions 24 and 2766 had significantly higher abundance of T3 and T4 precursor than the other iodination sites quantitated in this investigation, suggesting that those are the two primary sites of hormone synthesis.

## Discussion

The initial goal of this study was to develop an iodination map that could guide future studies of endogenous Tg enriched from patient serum and fine-needle aspirate samples. Thus, maximizing protein sequence coverage was imperative. This goal was achieved through the use of 3 different enzymes and high-performance LC-MS/MS instrumentation. For reference, the greater than 90% sequence coverage achieved in this work was slightly better than the 89% Dedieu et al reported [31], but their isolation process, procedure, instrumentation, and organism were different. The complementary nature of the different enzymes can be seen throughout the data presented herein, such as the percentage sequence coverage data presented in Table 1, where the individual enzymes yielded percentage sequence coverages of less than 81%, but when used in combination the percentage sequence coverage was greater than 90%. Additionally, unique iodination sites were identified with each enzyme as can be seen in Supplementary Tables S2 to S4 [36]. The identification of 54 iodinated tyrosine residues in this work is substantially more than that observed in other publications, suggesting that previous work may have been limited by the sensitivity of the respective techniques [30-32].

In this investigation, T3 and T4 precursors were identified in the well-established hormone synthesis positions. Hormone precursors were detected at positions Y24, Y1310, Y2573, and 2766 (see Table 2) in this investigation and relevant publications by Lamas and et al [30] and Coscia and colleagues [32]. In fact, Coscia et al [32] state that these are the only sites substantially contributing to hormone synthesis. The quantitative results in Fig. 3 suggest that the majority of the thyroid hormones are produced at the tyrosine residues in position 24 and 2766, but these results must be corroborated by additional studies from authentic patient samples. Additionally, hormone precursors were identified using discovery experiments in positions Y2563, 2564, Y2670, Y2672 using tryptic digestion of the Cell Sciences source material. To the best of our knowledge, these sites had not previously been established as hormone synthesis sites, but determining the extent to which they contribute substantially to overall hormone production will require more expansive quantitative studies than those presented herein. Lamas and colleagues [30] also detected T3 and T4 precursors in position Y704. However, in this study only monoiodinated and diiodinated tyrosine modifications were detected at this site, which aligns with the statements by Coscia and et al [32] and the mouse work conducted by Dedieu et al [31]. Additionally, T3 and T4 precursors were not detected in the targeted quantitative experiments performed in this investigation. Thus, given that this position has not been shown in this or other recent studies to produce hormone precursors, it is likely that the observation by Lamas and colleagues [30] was an artifact of the techniques used in that work and this site is unlikely to significantly contribute to hormone synthesis.

Historically, identification of tyrosyl donor products from thyroid hormone synthesis has proven challenging, with the chemical structure of donor residue after transfer remaining controversial. The two predominantly proposed products are dehydroalanine [37-39] or pyruvate [40-42], which results in a subsequent cleavage of the protein, although the mechanism of this reaction is inadequately understood. In this investigation, both potential products were detected, with 4 different dehydroalanine residues (see Table 3) and ` pyruvate product identified. In 1997, Gentile et al [43] identified dehydroalanine in bovine Tg using MS. More recently, Dedieu et al [31] did not identify dehydroalanine modifications, but did identify two pyruvate products. Therefore, to the best of our knowledge this is the first study identifying both products in the same Tg analysis. The amino acid positions of the donor residues are also interesting. One of the 4 dehydroalanine residues and the pyruvate product identified herein align with those observed by Coscia and colleagues [32], whereas the other 3 have not been previously described; however, more work is required to identify the corresponding acceptor sites for these new donors.

It is noteworthy that a considerable difference in the extent of iodination was observed between the two materials used in this study. Despite similar purities and quantities of material used, the Cell Sciences material resulted in many more iodination and hormone-producing sites identified, as well as a higher abundance of iodinated residues measured during the targeted quantitation experiments. It is also noteworthy that the highest abundance iodination state measured in the Cell Sciences material was the T3 precursor at position 2766. This is unexpected based on typical circulating concentrations in the normal population, and it is unknown if this Tg was sourced from subject(s) with atypical clinical presentations that would explain this observation. This was not the case for the Millipore-Sigma Tg, thus replication in authentic patient samples is necessary to understand the significance and frequency of this finding. Overall, the manufacturers do not provide much information about their isolation processes, thus, the reason for the discrepancies noted here are unclear.

The purpose of this investigation was to produce a map of Tg iodination to guide future quantitative studies in authentic patient fine-needle aspirates and ultimately serum samples. To maximize coverage of the posttranslation modifications of Tg, purified materials were used at concentrations that greatly exceed normal circulating concentrations in plasma. The next step is to identify which of the identified iodination sites and states are most clinically relevant and develop a highly sensitive method that enables the use of this as a diagnostic and monitoring technique for thyroid cancer. Additionally, application of this technique for assessment of iodoprophylaxis and coinciding implications, such as autoimmunity, is a future goal.

### Conclusions

This investigation is the first analysis of iodination of human Tg using modern hybrid orbitrap LC-MS/MS technologies. The findings in this study largely align with those derived from other techniques and organisms. However, the sequence coverage and extent of iodination observed in this study surpasses the findings of other studies, bringing us one step closer to a comprehensive knowledge of iodinated residues within Tg. Similarly, the identification of the products of tyrosyl donor residues expands on previous work, but a thorough understanding of the totality of the donor residues is still needed. This is also the first investigation to apply established LC-MS/MS quantitation techniques to measure the abundance of the iodination states at hormone-producing residues. This study provides an excellent foundation for future studies of Tg iodination using LC-MS/MS, which has the potential to revolutionize thyroid cancer monitoring and assessment of Tg iodination status.

## Data Availability

Some or all data sets generated during and/or analyzed during the present study are not publicly available but are available from the corresponding author on reasonable request.

## Abbreviations

DTT: dithiothreitol
I: iodination
I2: diiodination
IAA: iodoacetamide
LC-MS/MS: liquid chromatography–tandem mass spectrometry
T3: triiodothyronine
T4: thyroxine
TFA: trifluoroacetic acid
Tg: thyroglobulin
